# Anti-proteinase 3 antineutrophil cytoplasmic antibody reflects disease activity and predicts the response to steroid therapy in ulcerative colitis

**DOI:** 10.1186/s12876-021-01903-5

**Published:** 2021-08-23

**Authors:** Yuki Aoyama, Tomoki Inaba, Sakuma Takahashi, Hisae Yasuhara, Sakiko Hiraoka, Takeshi Morimoto, Hugh Shunsuke Colvin, Masaki Wato, Midori Ando, Satoko Nakamura, Koichi Mizobuchi, Hiroyuki Okada

**Affiliations:** 1 Department of Gastroenterology, Kagawa Prefectural Central Hospital, 1-2-1 Asahi-machi, Takamatsu, Kagawa 760-8557 Japan; 2 Department of Gastroenterology, Mitoyo General Hospital, 708 Himehama, Toyohama-cho, Kan-onji, Kagawa 769-1695 Japan; 3 Department of Gastroenterology and Hepatology, Okayama University Graduate School of Medicine, Dentistry and Pharmaceutical Sciences, 2-5-1 Shikata-cho, Kita-ku, Okayama 700-8558 Japan; 4 Department of Clinical Epidemiology, Hyogo College of Medicine, 1-1 Mukogawa-cho, Nishinomiya, Hyogo 663-8501 Japan; 5 Department of Pathology, Kagawa Prefectural Central Hospital, 1-2-1 Asahi-machi, Takamatsu, Kagawa 760-8557 Japan

**Keywords:** Ulcerative colitis, Biomarkers, Antineutrophil cytoplasmic antibody, Steroids, Refractory, Colonoscopy

## Abstract

**Background:**

Serum anti-proteinase 3 antineutrophil cytoplasmic antibody (PR3-ANCA) is a disease-specific antibody against granulomatosis with polyangiitis. PR3-ANCA is a useful serological marker for disease severity in ulcerative colitis (UC). The purpose of this study was to investigate whether PR3-ANCA levels could also predict the success of induction therapy and to compare its performance against other markers, including serum CRP and fecal hemoglobin.

**Methods:**

This was a multicenter retrospective study. In total, 159 patients with active-phase UC underwent colonoscopy. Disease activity was measured using the Mayo endoscopic subscore (MES). PR3-ANCA positivity and the response to induction therapy, either 5-aminosalicylic acid or steroid, were assessed. PR3-ANCA, CRP, and fecal hemoglobin were measured during the active phase, and during clinical remission.

**Results:**

Eighty-five (53.5%) of 159 patients with active UC were positive for PR3-ANCA. PR3-ANCA titers were significantly higher in the group of patients with MES 3 compared to patients with MES 1 (*P* = 0.002) or MES 2 (*P* = 0.035). Steroid therapy was administered to 56 patients with a median partial Mayo score of 7 (5–9), which is equivalent to moderate-to-severe disease activity. PR3-ANCA positivity of non-responders to steroid therapy was significantly higher than that of responders (71.9% vs, 41.7%, *P* = 0.030), whereas CRP and fecal hemoglobin were not predictive of steroid response. Multivariate analysis demonstrated that PR3-ANCA positivity was associated with non-response to steroid therapy (odds ratio 5.19; 95% confidence interval, 1.54–17.5; *P* = 0.008). Of the 37 patients treated to clinical remission who were also positive for PR3-ANCA during the active phase, 27 had an MES of ≥ 1, and 10 patients had an MES of 0. In clinical remission, the proportion of patients with MES 0 in 17 patients whose PR3-ANCA became negative was significantly higher than that in 20 patients whose PR3-ANCA remained positive (47.1% vs. 10.0%, *P* = 0.023).

**Conclusions:**

PR3-ANCA not only serves as a marker of disease activity, but also predicts the failure of steroid therapy in moderate-to-severe UC.

*Trial registration*: This study was retrospectively registered in the UMIN Clinical Trials Registry System (000039174) on January 16, 2020.

## Background

Ulcerative colitis (UC) is a chronic refractory inflammatory bowel disease of unknown cause [1], and its treatment is determined according to disease extent and activity. The first-line therapy for patients with mild-to-moderate activity is 5-aminosalicylic acid (5-ASA). Corticosteroids are used for patients who are refractory to 5-ASA or who have moderate-to-severe activity. However, approximately half of patients are resistant to or dependent on corticosteroids [2]. Several predictors of non-response to steroids, including lower hemoglobin level, partial Mayo score [3], and disease duration [4], have been reported. However, none of these markers are capable of fully predicting the response to steroid therapy, suggesting room for improvement.

It has long been postulated that autoimmune mechanisms are involved in the pathophysiology of UC [1, 5, 6], although immune targets have not been identified. Recently, Kuwada et al. reported that autoantibodies against integrin αvβ6 may be a reliable diagnostic marker for UC [7]. In addition, an association between UC and autoimmune disorders has also been reported [8].

Serum antineutrophil cytoplasmic antibody (ANCA) is a general term for antineutrophil cytoplasmic autoantibodies. Clinically significant ANCAs include cytoplasmic ANCAs (or anti-proteinase 3 (PR3)-ANCA), which target PR3, and perinuclear ANCAs (or myeloperoxidase (MPO)-ANCA), which target the MPO antigen. PR3-ANCA is a highly specific biomarker for granulomatosis with polyangiitis [9] and is a highly specific biomarker for eosinophilic granulomatosis with polyangiitis or microscopic polyangiitis [10]. MPO-ANCA is reportedly useful for differentiating UC from Crohn’s disease because UC cases in Western countries are often positive for MPO-ANCA [11–13]. However, in Japan and other regions of Asia, UC cases are commonly positive for PR3-ANCA, but not MPO-ANCA [14, 15]. PR3-ANCA has recently been reported to be useful as a serological marker for evaluating disease severity in UC [15], but the clinical significance of PR3-ANCA in UC has not yet been fully assessed.

UC is diagnosed either histologically or by colonoscopy. Clinical remission has been the primary target of treatment; however, endoscopic remission (when endoscopy shows no mucosal inflammation) has recently been identified as the more appropriate target [16]. An endoscopic evaluation is desirable to evaluate disease activity accurately; however, significant physical and financial burdens make it difficult to perform frequent endoscopic examinations. Fecal hemoglobin and calprotectin have recently been reported to be useful for evaluating disease activity [17, 18]. However, a serological marker for the assessment of disease activity would be extremely beneficial in clinical practice. Furthermore, a marker of non-response to induction therapy could provide physicians with important information.

We conducted a retrospective study to investigate whether PR3-ANCA could be a marker for successful induction therapy with 5-ASA or steroid, and to compare the performance of PR3-ANCA against other markers, including serum CRP and fecal hemoglobin levels.

## Methods

### Study design and patients

We conducted a multicenter retrospective study at the Kagawa Prefectural Central Hospital and Mitoyo General Hospital. We measured PR3-ANCA levels at the time of diagnosis of UC or when treatment was changed to exclude intestinal lesions caused by vasculitis. The inclusion criteria of this study were patients with active UC who underwent colonoscopy and PR3-ANCA measurement within 1 week between April 2016 and March 2020. A total of 173 consecutive patients were included in this study. Key exclusion criteria included being aged below five years; having a complication that could cause PR3-ANCA or MPO-ANCA positivity, such as an autoimmune disease, chronic extraintestinal inflammatory disease [19], or a malignant tumor [20]; already being initiated on drug therapy, including steroids, biologics such as tumor necrosis factor-α inhibitor, tacrolimus, or Janus kinase inhibitor; or patients who declined to participate by opting out. We defined patients under the age of 17 as children. A certified rheumatologist confirmed that no patient had granulomatosis with polyangiitis during UC.

### Diagnosis and disease activity of UC

UC was diagnosed based on the Lennard–Jane criteria [21]. Disease types were classified based on the Montreal Classification [22] as proctitis, left-sided, and extensive. According to colonoscopy results, disease activity was evaluated by assigning the Mayo Endoscopic Subscore (MES) [23] to the sites with the most severely inflamed mucosa. An MES of 0 was defined as remission, and an MES ≥ 1 was defined as an active-phase disease. The pathological activity was evaluated by assigning the Matts grade [24] to the biopsy results of the site with the most severely inflamed mucosa. By agreement, without referring to past medical records, MES was assessed by two endoscopy specialists, and the Matts grade was determined by two pathologists.

Clinical activity was evaluated using the partial Mayo score, which consists of the frequency of defecation, rectal bleeding, and physician rating. A score of ≤ 1 was defined as clinical remission, and a score of 2–4, 5–7, and > 8 was defined as mild, moderate, and severe conditions in the active phase, respectively.

### Response to the induction therapy

We examined the response to induction therapy for active-phase colitis in each participant. Those who achieved clinical remission within 90–120 days after starting induction therapy were considered to be responders. Non-response to steroid therapy consisted of steroid resistance and steroid dependence. Steroid resistance is defined as an exhibition of no clinical improvement after treatment with high-dose oral steroids (40–60 mg/day prednisone or equivalent) within 30 days or no clinical improvement after treatment with high-dose intravenous prednisone within 7–10 days [25]. Steroid dependence is defined as an initial response to treatment with high-dose prednisone, but then relapse during tapering or shortly after drug discontinuation and requiring re-introduction to maintain symptom control [26].

### Measurement

PR3-ANCA and MPO-ANCA levels were measured at an external institution using a kit (STACIA® MEBLux™ test; MBL) that included a chemiluminescent enzyme immunoassay (CLEIA) on serum that was cryopreserved at − 10 °C or below. The detection limit values for PR3-ANCA and MPO-ANCA were 1.0 U/mL. The cut-off value for PR3-ANCA is 3.5 U/mL [15]. Serum C-reactive protein (CRP) (cut-off value, 0.14 mg/dL) and fecal hemoglobin (cut-off value, 100 ng/mL) were selected as activity indicators of the clinical test. Serum CRP levels were measured at the same time as PR3-ANCA, and stool samples for fecal hemoglobin were obtained within two or three days before colonoscopy. Cytomegalovirus (CMV) infection was defined as positive if either CMV immunostaining of biopsy tissue or blood CMV pp65 antigen (C7-HRP) was positive.

### Endpoints

The primary endpoint of this study was to investigate whether PR3-ANCA reflects the success of induction therapy. The secondary endpoint was to reveal the difference between PR3-ANCA and other inflammatory markers, such as serum CRP and fecal hemoglobin levels.

### Statistical analysis

We described the characteristics of patients with the number and percentage for categorical variables and mean with standard deviation or median (range) for continuous variables according to the distribution. We used Fisher’s exact test or the Chi-squared test for categorical variables and the Mann–Whitney U test or Student’s t-test for continuous variables. We used Dunnett’s test for comparisons of PR3-ANCA among MES using MES 3 as a reference, and the same comparisons were performed for CRP and fecal hemoglobin. Spearman’s rank correlation was also used for correlations between UC activity and serum PR3-ANCA, serum CRP, and fecal hemoglobin levels. Multivariate logistic regression analysis was used to clarify the factors associated with non-response to steroid therapy. The level of statistical significance was set at a two-sided *P* value of < 0.05. EZR (version 3.4.1, Windows) was used for the statistical analyses.

## Results

Of the 173 patients with UC, 159 were analyzed. Of the patients excluded, two were already being treated with steroids, and 12 had either an autoimmune disease, chronic extraintestinal inflammatory disease, or a malignant tumor. Of the 159 patients, 41 (25.8%) presented with an initial attack of colitis. Patients included 80 men and 79 women with a mean age of 43.5 years (± 17.2 years). The mean age of onset was 36.6 years (± 16.5 years), and the median disease duration was 4.0 years (1.0–11.0). Seven children aged 11, 13,13, 15, 16, 16, and 16 were included in the study. There were 107 cases of extensive colitis (67.3%), 39 cases of left-sided colitis (24.5%), and 13 cases of proctitis (8.2%) (Table 1). Endoscopic images showed classic UC findings, and biopsy results showed no evidence of vasculitis. Thirty-five patients had an MES of 1, 49 had an MES of 2, and 75 had an MES of 3.

**Table 1:** Characteristics of patients

	n = 159
Age, mean ± SD (years)	43.5 ± 17.2
Sex, male/female	80 (50.3%)/79 (49.7%)
BMI, mean ± SD (kg/m^2^)	22.4 ± 3.5
History of smoking	52 (32.7%)
Current alcohol consumption	41 (25.8%)
Age at onset, mean ± SD (years)	36.6 ± 16.5
Disease duration, median (range), years	4.0 (1.0–11.0)
Initial attack	41 (25.8%)
*Type*	
Extensive	107 (67.3%)
Left-sided	39 (24.5%)
Proctitis	13 (8.2%)
Frequency of defecation per day, median (range)	5 (3–8)
Bloody stool	95 (59.7%)
CRP, median (range), mg/dL	0.19 (0.05–1.31)
Alb, median (range), g/dL	4.1 (3.7–4.4)
Plt, median (range), × 10^4^/μL	30.0 (24.4–38.4)
Fecal hemoglobin, median (range), ng/mL	1559 (144–5284)
CMV infection	9 (5.7%)
MES, median (range)	2 (1–3)
Matts grade, median (range)	4 (3–5)

SD, standard deviation; BMI, body mass index; CRP, C-reactive protein; Alb, albumin; Plt, platelet; CMV, cytomegalovirus; MES, Mayo Endoscopic Subscore

Table 2 shows patients’ PR3-ANCA or MPO-ANCA levels grouped by MES. Of the 159 patients, 85 (53.5%) had serum PR3-ANCA ≥ 3.5 U/mL, indicating the PR3-ANCA-positive group, and 74 (46.5%) had serum PR3-ANCA < 3.5 U/mL, indicating the negative group. The PR3-ANCA-positive group had significantly higher MES and pathological activity (*P* = 0.032 and 0.013, respectively) than the negative group (Table 3). In seven child patients, three were PR3-ANCA-positive and none were MPO-ANCA-positive.

**Table 2:** Patients’ PR3-ANCA and MPO-ANCA levels by MES group

MES	1 (n = 35)	2 (n = 49)	3 (n = 75)
PR3-ANCA (≥ 3.5)	14	24	47
PR3-ANCA (< 3.5)	21	25	28
MPO-ANCA (≥ 1.0)	2	4	4
MPO-ANCA (< 1.0)	33	45	71

MES, Mayo Endoscopic Subscore; PR3-ANCA, anti-proteinase 3 antineutrophil cytoplasmic antibody; MPO-ANCA, myeloperoxidase antineutrophil cytoplasmic antibody

**Table 3:** Comparison of the PR3-ANCA-positive and negative groups in the active phase

	PR3-ANCA positive (n = 85)	PR3-ANCA negative (n = 74)	*P* value
Age, mean ± SD (years)	40.8 ± 17.2	46.6 ± 16.7	0.034
Sex, male/female	45 (52.9%)/40 (47.1%)	39 (52.7%)/35 (47.3%)	1
BMI, mean ± SD (kg/m^2^)	22.4 ± 3.2	22.3 ± 3.7	0.89
History of smoking	24 (28.2%)	28 (37.8%)	0.24
Current alcohol consumption	22 (25.9%)	19 (25.7%)	1
Age at onset, mean ± SD (years)	35.3 ± 16.9	38.2 ± 16.0	0.27
Disease duration, median (range), years	3.0 (0–8)	6.0 (1–13)	0.083
*Type*			0.28
Extensive	62 (72.9%)	45 (60.8%)	
Left-sided	17 (20.0%)	22 (29.7%)	
Proctitis	6 (7.1%)	7 (9.5%)	
Frequency of defecation per day, median (range)	5 (3–8)	4 (2–7)	0.11
Bloody stool	54 (63.5%)	41 (55.4%)	0.33
CRP, median (range), mg/dL	0.32 (0.06–1.39)	0.15 (0.04–0.87)	0.15
Alb, median (range), g/dL	4.0 (3.2–4.4)	4.2 (3.8–4.4)	0.089
Plt, median (range), × 10^4^/μL	31.2 (25.4–40.2)	29.9 (22.6–35.5)	0.20
Fecal hemoglobin, median (range), ng/mL	1703 (264–6054)	1158 (137–4675)	0.278
CMV infection	5 (5.9%)	4 (5.4%)	1
MES, median (range)	3 (2–3)	2 (1–3)	0.032
Matts grade, median (range)	4 (3–5)	3 (3–4)	0.013

SD, standard deviation; BMI, body mass index; CRP, C-reactive protein; Alb, albumin; Plt, platelet; CMV, cytomegalovirus; MES, Mayo Endoscopic Subscore

### Relationship between MES versus CRP and fecal hemoglobin

**Fig. 1:**
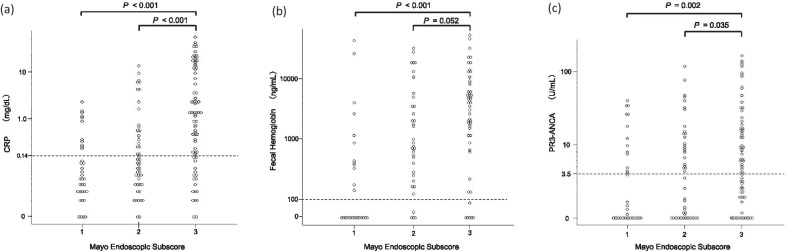
Correlation between MES versus serum CRP levels (**a**), fecal hemoglobin levels (**b**), and serum PR3-ANCA levels (**c**). MES, Mayo Endoscopic Subscore; CRP, C-reactive protein; PR3-ANCA, anti-proteinase 3 antineutrophil cytoplasmic antibody

The median CRP titers at MES 1, 2, and 3 were 0.05 (0.03–0.21), 0.11 (0.04–0.4), and 0.83 (0.15–5.9), respectively. There was a statistically significant difference in CRP titers between MES 3 and 2, or MES 3 and 1 (both *P* < 0.001). A positive correlation was observed between the MES and CRP levels (r = 0.444; *P* < 0.001) (Fig. 1a).

To assess the fecal hemoglobin levels, 115 patients were analyzed. Of 159 participants, 44 (27.7%) could not provide stool samples because of sampling difficulty in the active phase of the disease. The median fecal hemoglobin titers at MES 1, 2, and 3 were 0 (0–417), 853 (270–4290), and 4031 (1173–8090), respectively. There was a statistically significant difference in fecal hemoglobin titer between MES 3 and 1 (*P* < 0.001), but there was no significant difference between MES 3 and 2 (*P* = 0.052). A positive correlation was observed between MES and the fecal hemoglobin level (r = 0.469; *P* < 0.001) (Fig. 1b).

### Relationship between MES or Matts grade versus PR3-ANCA

The median PR3-ANCA titers at MES 1, 2, and 3 were 1.1 (0–7.6), 3.4 (1.0–10.1), and 6.1 (2.1–18.1), respectively. There was a statistically significant difference in PR3-ANCA titers between MES 3 and 1 (*P* = 0.002) and between MES 3 and 2 (*P* = 0.035). A positive correlation was observed between MES and serum PR3-ANCA levels (r = 0.228; 95% confidence interval [CI], 0.075–0.371; *P* = 0.004) (Fig. 1c).

Of the 159 patients, eight had a Matts grade of 1 (5.0%), 20 had a Matts grade of 2 (12.6%), 43 had a Matts grade of 3 (27.1%), 36 had a Matts grade of 4 (22.6%), and 52 had a Matts grade of 5 (32.7%). Titers of PR3-ANCA at Matts grade 1, 2, 3, 4, and 5 were 0 (0–3.2), 1.4 (0–10.5), 4.1 (0–22.5), 3.3 (0.8–8.4), and 6.3 (2.4–16.8), respectively. A positive correlation was observed between the Matts grade and serum PR3-ANCA levels (r = 0.234; *P* = 0.003).

### Relationship between PR3-ANCA versus CRP and fecal hemoglobin

No positive correlation was observed between PR3-ANCA and CRP levels (r = 0.152; *P* = 0.056). CRP positivity in 85 PR3-ANCA-positive patients was not different from that of 74 PR3-ANCA-negative patients (53% vs. 38%, *P* = 0.199). No positive correlation was observed between PR3-ANCA and fecal hemoglobin (r = 0.17; *P* = 0.071). Fecal hemoglobin positivity in 85 PR3-ANCA-positive patients was the same as that in 74 PR3-ANCA-negative patients (57.6% vs. 54.1%, *P* = 0.749).

### PR3-ANCA as a predictor of non-response to the induction therapy

The induction therapy response for active-phase colitis in each participant was assessed 98 days after starting the treatment. The assessment was performed between day 91 and 112. Therapy with 5-ASA was introduced in 69 patients with a median partial Mayo score of 5 (3–6). Steroid therapy was introduced in 56 patients with a median partial Mayo score of 7 (5–9).

**Fig. 2:**
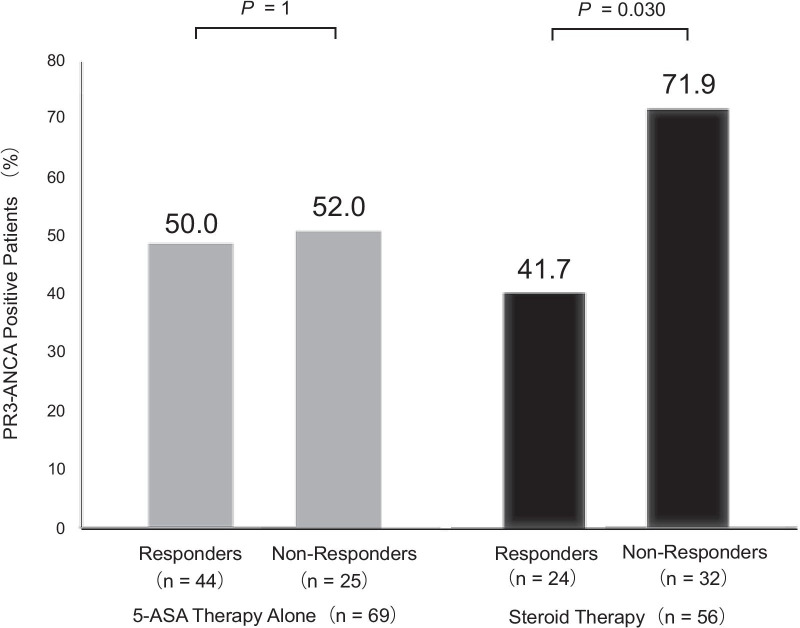
PR3-ANCA positivity in responders and non-responders to induction therapy. 5-ASA, 5-aminosalicylic acid; PR3-ANCA, anti-proteinase 3 antineutrophil cytoplasmic antibody

Figure 2 shows PR3-ANCA positivity in responders and non-responders to treatment with 5-ASA or steroid therapy. The proportion of PR3-ANCA positivity was not significantly different with respect to the response to 5-ASA therapy (50% of responders vs. 52% of non-responders, *P* = 1). PR3-ANCA positivity in non-responders to steroid therapy was significantly higher than that in responders (71.9% vs. 41.7%, *P* = 0.030). PR3-ANCA positivity in non-responders to steroid therapy remained high, even after the exclusion of four child patients (72.4% vs. 43.5%, *P* = 0.048).

CRP positivity of non-responders to steroid therapy was not different from that of responders (78.1% vs. 58.3%, *P* = 0.146). Fecal hemoglobin positivity of non-responders to steroid therapy was not different from that of responders (65.6% vs. 54.2%, *P* = 0.42).

Table 4 shows the comparison between responders and non-responders to 5-ASA therapy. The median partial Mayo scores of responders and non-responders were 4 (3–6) and 5 (4–6), respectively, without a significant difference (*P* = 0.55). The median PR3-ANCA titers in responders and non-responders to 5-ASA therapy were 2.5 (1.4–14.8) and 3.9 (1.7–14.2), respectively, without a statistically significant difference (*P* = 0.20). Of the non-responders to 5-ASA therapy, the proportions of those who were PR3-ANCA-positive were 50.0% (2/4) who were MES 1, 28.6% (2/7) who were MES 2, and 64.3% (9/14) who were MES 3.

**Table 4:** Comparison of responders and non-responders to 5-ASA therapy

	5-ASA responders (n = 44)	5-ASA non-responders (n = 25)	*P* value
Age, mean ± SD (years)	42.8 ± 16.8	49.0 ± 17.6	0.16
Sex, M/F	24 (54.5%)/20 (45.5%)	12 (48.0%)/13 (52.0%)	0.625
BMI, mean ± SD (kg/m^2^)	21.7 ± 2.5	22.2 ± 3.1	0.49
History of smoking	17 (38.6%)	5 (20.0%)	0.18
Current alcohol consumption	13 (29.5%)	8 (32.0%)	1
Age at onset, mean ± SD (years)	35.1 ± 14.8	42.8 ± 17.2	0.064
Disease duration, median (range), years	3.0 (1–12)	3.0 (1–8)	0.55
*Type*			1
Extensive	24 (54.5%)	15 (60.0%)	
Left-sided	15 (34.1%)	8 (32.0%)	
Proctitis	5 (11.4%)	2 (8.0%)	
Frequency of defecation per day, median (range)	4 (1–6)	5 (4–8)	0.046
Bloody stool	20 (45.5%)	19 (76.0%)	0.022
CRP, median (range), mg/dL	0.08 (0.04–0.33)	0.57 (0.09–2.8)	0.011
Alb, median (range), g/dL	4.3 (4.1–4.5)	3.9 (3.2–4.1)	< 0.001
Plt, median (range), × 10^4^/μL	29.5 (22.5–33.4)	31.4 (26.9–36.8)	0.19
Fecal hemoglobin, median (range), ng/mL	710 (122–3938)	5608 (676–14,270)	0.012
CMV infection	0 (0%)	1 (25.0%)	0.36
MES, median (range)	2 (1–3)	3 (2–3)	0.005
Matts grade, median (range)	3 (2–4)	4 (4–5)	< 0.001
Partial Mayo score	4 (3–6)	5 (4–6)	0.55
PR3-ANCA positive	21 (47.7%)	13 (52.0%)	0.81
PR3-ANCA, median (range), U/mL	2.5 (1.4–14.8)	3.9 (1.7–14.2)	0.20

BMI, body mass index; CRP, C-reactive protein; Alb, albumin; Plt, platelet; PR3-ANCA, anti-proteinase 3 anti-neutrophil cytoplasmic antibody; MPO-ANCA, myeloperoxidase anti-neutrophil cytoplasmic antibody; CMV, cytomegalovirus; MES, Mayo endoscopic subscore

Table 5 shows the comparison between responders and non-responders to steroid therapy. The median partial Mayo scores of 24 responders and 32 non-responders were 6 (5–7) and 7 (5–9), respectively, without a significant difference (*P* = 0.45). The median PR3-ANCA titers in responders and non-responders to steroid therapy were 1.9 (1.2–7.5) and 8.9 (1.9–25.5), respectively, with a statistically significant difference (*P* = 0.041). Of the non-responders to steroid therapy, the percentage of those who were PR3-ANCA positive was 0% (0/1) for MES 1, 83.3% (5/6) for MES 2, and 76.0% (18/25) for MES 3.

**Table 5:** Comparison of responders and non-responders to steroid therapy

	Steroid responders (n = 24)	Steroid non-responders (n = 32)	*P* value
		14 steroid-resistant 18 steroid-dependent	
Age, mean ± SD (years)	46.1 ± 17.8	43.5 ± 19.8	0.606
Sex, M/F	15 (62.5%)/9 (37.5%)	16 (50.0%)/16 (50.0%)	0.421
BMI, mean ± SD (kg/m^2^)	23.3 ± 4.6	23.0 ± 3.9	0.83
History of smoking	9 (37.5%)	11 (34.4%)	1
Current alcohol consumption	6 (25.0%)	8 (25.0%)	1
Age at onset, mean ± SD (years)	38.9 ± 17.7	37.8 ± 19.9	0.82
Disease duration, median (range), years	6.0 (0–9)	3.5 (1–9.5)	0.71
*Type*			1
Extensive	20 (83.3%)	27 (84.4%)	
Left-sided	4 (16.7%)	5 (15.6%)	
Proctitis	0 (0%)	0 (0%)	
Frequency of defecation per day, median (range)	5 (3–8)	8 (5–12)	0.017
Bloody stool	11 (45.8%)	27 (84.4%)	0.004
CRP, median (range), mg/dL	0.29 (0.05–2.1)	1.18 (0.35–6.0)	0.074
Alb, median (range), g/dL	4.1 (3.6–4.4)	3.4 (2.7–4.1)	0.006
Plt, median (range), × 10^4^/μL	29.9 (21.8–37.8)	39.0 (28.3–45.6)	0.066
Fecal hemoglobin, median (range), ng/mL	999 (50–5664)	3630 (1329–9417)	0.074
CMV infection	2 (8.3%)	3 (9.4%)	1
MES, median (range)	3 (2–3)	3 (3–3)	0.081
Matts grade, median (range)	4 (3–5)	5 (4–5)	0.015
Partial Mayo score	6 (5–7)	7 (5–9)	0.45
PR3-ANCA positive	10 (41.7%)	23 (71.9%)	0.030
PR3-ANCA, median (range), U/mL	1.9 (1.2–7.5)	8.9 (1.9–25.5)	0.041

BMI, body mass index; CRP, C-reactive protein; Alb, albumin; Plt, platelet; PR3-ANCA, anti-proteinase 3 anti-neutrophil cytoplasmic antibody; MPO-ANCA, myeloperoxidase anti-neutrophil cytoplasmic antibody; CMV, cytomegalovirus; MES, Mayo Endoscopic Subscore

Among 32 non-responders to steroid therapy, the median PR3-ANCA titers in 14 steroid-resistant and 18 steroid-dependent patients were 11.0 (6.7–51.3) and 6.1 (1.2–12.0), respectively. PR3-ANCA titers in steroid-resistant patients were higher than those in steroid-dependent patients, but the difference was not statistically significant (*P* = 0.105).

### Performance of PR3-ANCA for predicting a non-response to steroid therapy

Univariate analysis revealed significant differences between responders and non-responders to steroid therapy in the frequency of defecation (median [range]: 5 [3–8] vs. 8 [5–12], *P* = 0.017), bloody stool (45.8% [n = 11] vs. 84.4% [n = 27], *P* = 0.004), albumin titers (4.1 [3.6–4.4] vs. 3.4 [2.7–4.1], *P* = 0.006), Matts grade (4 [3–5] vs. 5 [4, 5], *P* = 0.015), PR3-ANCA positivity (41.7% [n = 10] vs. 71.9% [n = 23], *P* = 0.030), and PR3-ANCA titers (1.9 [1.2–7.5] vs. 8.9 [1.9–25.5], *P* = 0.041) (Table 5).

Multivariate analysis using logistic regression analysis showed that PR3-ANCA positivity was significantly associated with a non-response to steroid therapy (odds ratio 5.19, 95% CI: 1.54–17.5; *P* = 0.008). No other factors, including CRP, fecal hemoglobin, disease duration, and partial Mayo score, were significantly associated with a non-response to steroid therapy (Table 6).

**Table 6:** The association between a non-response to steroid therapy and explanatory variables

	Odds ratio	95% Confidence interval	*P* value
Disease duration	1.01	0.92–1.10	0.871
CRP	0.97	0.81–1.15	0.693
Fecal hemoglobin	1.00	1.00–1.00	0.804
Partial Mayo score	1.35	0.97–1.87	0.077
PR3-ANCA positive	5.19	1.54–17.50	0.008

CRP, C-reactive protein; PR3-ANCA, anti-proteinase 3 anti-neutrophil cytoplasmic antibody

### Change of PR3-ANCA in responders and non-responders to steroid therapy

Of the 24 responders to steroid therapy, 10 patients (41.7%) were initially positive for PR3-ANCA and were successfully treated for clinical remission with steroids. The median titer of PR3-ANCA-positive responders to steroids was 14.1 (8.2–19.0) during the active phase, and became significantly lower at 1.5 (1.0–4.2) in clinical remission (*P* = 0.002). In 70.0% (7/10) of PR3-ANCA-positive responders, PR3-ANCA became negative in clinical remission.

Additional therapy was administered to 32 non-responders to steroid therapy. Among these patients, 23 (71.9%) were PR3-ANCA positive. Nineteen patients (82.6%) of these 23 PR3-ANCA-positive non-responders were successfully treated for clinical remission. Biologics, including tumor necrosis factor-α inhibitor, reinduction of high-dose steroids, tacrolimus, and Janus kinase inhibitor with leukocytapheresis (LCAP), achieved clinical remission in 11 of 13 patients (84.6%), in five of seven patients (71.4%), in two of two patients (100%), and in one of one patient (100%), respectively.

The median titer of PR3-ANCA in PR3-ANCA-positive non-responders was 23.0 (7.6–69.2) in the active phase and 6.7 (2.0–32.1) in clinical remission. The reduction in PR3-ANCA levels was significant when comparing the active phase and clinical remission (*P* < 0.001). However, PR3-ANCA became negative in only 36.8% (7/19) of PR3-ANCA-positive non-responders during clinical remission.

### Changes in PR3-ANCA, CRP, or fecal hemoglobin levels in patients who underwent follow-up colonoscopy in clinical remission

Fifty-eight patients (active phase PR3-ANCA-positive cases) were observed for 7.6 months (5.3–14.7) and underwent endoscopic examination and measurements of PR3-ANCA, CRP, and fecal hemoglobin. Clinical remission was achieved in 37 of the 58 cases (63.8%), and the relationships between MES, which was assessed in clinical remission versus serum PR3-ANCA, serum CRP, and fecal hemoglobin levels, were analyzed in these 37 patients (Table 7). Twenty-seven patients had an MES ≥ 1, and only 10 patients had an MES of 0.

**Table 7:** PR3-ANCA, CRP, and fecal hemoglobin levels by MES group in clinical remission cases

MES	0 (n = 10)	1 (n = 9)	2 (n = 16)	3 (n = 2)
PR3-ANCA positive, (n = 20)	2	5	11	2
PR3-ANCA negative, (n = 17)	8	4	5	0
CRP positive, (n = 10)	0	3	7	0
CRP negative, (n = 27)	10	6	9	2
FH positive, (n = 15)	1	2	11	1
FH negative, (n = 18)	7	6	5	0

MES, Mayo Endoscopic Subscore; PR3-ANCA, anti-proteinase 3 antineutrophil cytoplasmic antibody; CRP, C-reactive protein; FH, fecal hemoglobin

For cases with MES 0, seven were treated with steroids, two were treated with biologics, and one was treated with tacrolimus as induction therapy. For MES ≥ 1 patients, 12 were treated with 5-ASA, 10 were treated with steroids, two were treated with biologics, two were treated with tacrolimus, and one was treated with 5-ASA plus LCAP as induction therapy. During the observation period, maintenance therapy was almost the same in MES 0 and MES ≥ 1 patients.

**Fig. 3:**
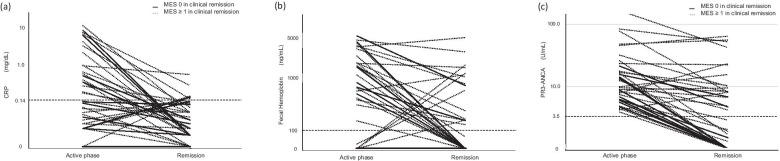
Changes in CRP titers (**a**), fecal hemoglobin titers (**b**), and PR3-ANCA titers (**c**) in cases that reached clinical remission. Significant reductions in the levels were observed in cases with MES 0 and MES ≥ 1 for fecal hemoglobin and PR3-ANCA when comparing the active phase and clinical remission (*P* = 0.036 and *P* = 0.015, *P* < 0.001 and *P* = 0.043, respectively). CRP, C-reactive protein; PR3-ANCA, anti-proteinase 3 antineutrophil cytoplasmic antibody; MES, Mayo Endoscopic Subscore

The reductions in CRP levels in cases with MES 0 and MES ≥ 1 were not significant when comparing the active phase and clinical remission (*P* = 0.148 and *P* = 0.098, respectively) (Fig. 3a). The reductions in fecal hemoglobin levels in cases with MES 0 and MES ≥ 1 were significant when comparing the active phase with clinical remission (*P* = 0.036 and *P* = 0.015, respectively) (Fig. 3b). The reductions in PR3-ANCA levels in cases with MES 0 and MES ≥ 1 were significant when comparing the active phase and clinical remission (*P* < 0.001 and *P* = 0.043, respectively) (Fig. 3c). For cases with MES 0, the median titer of PR3-ANCA was 7.8 (6.1–14.0) in the clinical active phase and 0 (0–1.4) in clinical remission. For MES ≥ 1 patients, the median titer of PR3-ANCA was 12.3 (7.6–24.7) in the clinical active phase and 8.1 (2.0–12.9) in clinical remission.

In clinical remission, the fecal hemoglobin in 33 patients became negative in 18 patients and remained positive in 15 patients. The ratio of MES 0 in 18 patients whose fecal hemoglobin became negative was higher than that in 15 patients whose fecal hemoglobin remained positive with a statistically significant difference (38.9% vs. 6.7%, *P* = 0.046).

### Significance of PR3-ANCA as a target for treatment

Although clinical remission was achieved in 37 cases, PR3-ANCA was negative in 17 patients and remained positive in 20 patients. The ratio of MES 0 in the 17 patients whose PR3-ANCA became negative was higher than that in the 20 patients whose PR3-ANCA remained positive with a statistically significant difference (47.1% vs 10.0%, *P* = 0.023). Concerning detectability of the mucosal healing equivalent to MES 0, PR3-ANCA had a sensitivity of 66.7%, a specificity of 80.0%, a positive predictive value of 90.0%, a negative predictive value of 47.1%, and a proper diagnosis rate of 70.3%.

## Discussion

In this study, UC patients who were non-responders to steroid therapy had higher positive rates and values for PR3-ANCA than responders. In contrast, the positivity of CRP and fecal hemoglobin in non-responders to steroid therapy was not different from that of responders. Multivariate analysis using logistic regression analysis demonstrated that PR3-ANCA positivity was associated with non-response to steroid therapy (odds ratio 5.19). Concerning the inflammatory markers, PR3-ANCA, CRP, and fecal hemoglobin were correlated with disease activity based on MES in the active phase. However, a positive correlation was not observed between PR3-ANCA and CRP levels and fecal hemoglobin. PR3-ANCA and fecal hemoglobin levels reflected endoscopic activity in clinical remission.

CRP is primarily dependent on liver production induced by circulating interleukin 6, and only modest to absent CRP responses are observed in UC, despite active inflammation in the colon [27]. In this study, the usefulness of CRP as a marker of disease activity was limited. Fecal calprotectin is commonly used to evaluate disease activity in Western countries. However, we used fecal hemoglobin as a marker in this study because fecal calprotectin was not available in Japan for clinical use. Fecal hemoglobin is a useful marker of disease activity in UC, which directly reflects the level of mucosal damage of the colon [17] and reflects the inflammation of the mucosa in this study.

Several serologic markers for disease activity of UC, such as trefoil factor 3 [28] and leucine-rich alpha-2 glycoprotein (LRG) [29], have been reported. LRG has recently become available in Japan for clinical use, but is affected by several inflammatory factors outside the intestine [30]. Further study is needed to investigate the significance of LRG in the management of patients with UC.

The positivity rate for PR3-ANCA in UC has been reported to be 39.2% among Japanese individuals by Takedatsu et al. [15]. In addition, this value is reportedly 8.6% among Chinese individuals, 12.1% among Swedish individuals [31], and 14.1% among Spanish individuals [32]. In the present study considering Japanese individuals, the PR3-ANCA positivity rate was 53.5%, which is close to that reported by Takedatsu et al., and higher than that reported in other countries.

Measurements of PR3-ANCA have been known to yield different results depending on the method used. Methods of measuring ANCA can be broadly classified as indirect immunofluorescence (IIF) and enzyme immunoassays (EIA). IIF is reportedly unreliable because of changes in antigenicity or admixture of other antigens [33]. EIA methods include enzyme-linked immunosorbent assays (ELISAs) and CLEIAs. Regarding EIA, CLEIA is reportedly more sensitive than ELISA for the examination of the same samples [34]. Although PR3-ANCA was measured using the same CLEIA method in the present study and by Takedatsu et al. [15], other studies have not described the measurement methods in detail. The differences in the positivity rates of previous studies may be the result of differences in the measurement methods used.

Factors that affect the PR3-ANCA positivity rate in UC include patient background and UC activity. Important patient background characteristics include age and concomitant disease. A higher positive rate of PR3-ANCA, such as 57.6% [35] compared to that of previous studies on adult patients, and the usefulness for differential diagnosis of Crohn’s disease [36] have been reported in children with UC. Further studies are necessary to investigate whether PR3-ANCA predicts a non-response to steroid therapy in children. However, the small number of children included in this study had little effect on the results.

A relationship between PR3-ANCA and other diseases including chronic respiratory disease and malignant tumors has also been reported [19, 20]. Previous investigations of the relationship between UC and PR3-ANCA have not considered factors other than UC that could lead to PR3-ANCA-positivity. The present study excluded cases involving complications that could affect PR3-ANCA.

The relationship between UC activity and PR3-ANCA remains controversial. PR3-ANCA-positive UC has been reported to involve a few refractory cases as well as those severe enough to require surgery or intervention with immunoregulators or biologics [34]. However, there have been reports of refractory instances and those that involve primary sclerosing cholangitis as a complication [37]. Furthermore, Takedatsu et al. [15] recently reported that PR3-ANCA is related to UC activity.

Although the underlying cause of UC remains unknown, large quantities of neutrophils are observed at the inflamed sites of the intestinal mucosa. No prior study has investigated how PR3-ANCA affects inflammation of the mucosa caused by UC. Hyperactivity of neutrophils due to the formation and release of neutrophil extracellular traps (NETs) has been discussed as it pertains to the pathology of PR3-ANCA-associated vasculitis [10, 38, 39]. NETs are a network-like structure inside neutrophils with a mixture of neutrophil intranuclear DNA, neutrophil cytoplasmic MPO and PR3, neutrophil elastase, bactericidal/permeability-increasing protein, lactoferrin, and other antimicrobial proteins. In response to infection stimuli, NETs are released outside the cell, trapping and killing pathogens while minimizing host cell death [40]. However, NETs directly injure the vascular endothelial cells. Therefore, they are strictly controlled by being broken down, mainly by deoxyribonuclease I [41]. PR3-specific mouse monoclonal antibodies have been shown to assist in the formation of NETs [39]. Overproduction of NETs has been inferred to lower immune tolerance to MPO and PR3, consequently causing ANCA overproduction, which induces neutrophil hyperactivity and promotes inflammation [39, 42–44].

Of the patients in the current study with mild colitis treated with 5-ASA as induction therapy, PR3-ANCA positivity in responders and non-responders was no different at approximately 50% in the two groups. PR3-ANCA is produced regardless of the degree of inflammation in UC. However, in moderate-to-severe colitis patients treated with steroids as induction therapy, PR3-ANCA positivity in non-responders was significantly higher than that in responders. Therefore, when inflammation becomes advanced, PR3-ANCA may be more strongly involved in aggravating the inflammatory process and diminishing the therapeutic effect of the drugs; however, the underlying mechanism remains unclear.

ANCA-associated vasculitis is treated with a combination therapy comprising of steroids and immunoregulators [45, 46]. With PR3-ANCA-positive UC, PR3-ANCA may intensify inflammation of the mucosa caused by UC, thus, rendering steroid monotherapy insufficient to treat moderate-to-severe UC. If this is the case, then PR3-ANCA could provide treating physicians with useful information when considering treatment options other than steroid monotherapy. In addition, inhibiting PR3-ANCA may be useful as a goal for induction therapy.

This study has several limitations. First, this was a retrospective study with a short follow-up period and a relatively small sample size. Ten responders and 23 non-responders to steroid therapy were PR3-ANCA-positive, and this small sample size may have been insufficient for multivariate analysis. Second, the observational study could not exclude the effects of other possible confounding factors on non-response to steroid therapy. Third, we made many comparisons within a limited sample size. Although we conducted these analyses based on the pre-determined hypothesis, such tests should be interpreted cautiously. Fourth, a single method was used to measure serum PR3-ANCA levels. Other measurement methods must be used to investigate whether the results are similar. Fifth, because all patients included in this study were Japanese, it is unclear whether our findings can be applied to other populations. To overcome these limitations, it is necessary to investigate the relationship between PR3-ANCA and the effects of several induction therapies in a large prospective study across multiple ethnic groups. Despite these limitations, this is the first study to demonstrate that PR3-ANCA can serve as a predictor of non-response to steroid therapy in patients with UC. The results obtained in this study encourage researchers to investigate the mechanism by which PR3-ANCA aggravates inflammation in UC.

## Conclusions

Approximately half of active-phase UC cases were positive for serum PR3-ANCA and were predictive of more severe disease as well as failure of steroid therapy. PR3-ANCA may exacerbate inflammation of the mucosa in UC, and inhibiting PR3-ANCA may be useful as a strategy for induction therapy. We have launched a prospective study to clarify the relationship between the efficacy of different induction therapies and PR3-ANCA, and the relationship between the relapse of UC and PR3-ANCA.

## Data Availability

The datasets generated and/or analyzed during the current study are available from the corresponding author upon reasonable request.

## Abbreviations

ANCA: Antineutrophil cytoplasmic antibody
CI: Confidence interval
CLEIA: Chemiluminescent enzyme immunoassays
CMV: Cytomegalovirus
CRP: C-reactive protein
EIA: Enzyme immunoassay
ELISA: Enzyme-linked immunosorbent assays
IIF: Indirect immunofluorescence
LCAP: Leukocytapheresis
LRG: Leucine-rich alpha-2 glycoprotein
MES: Mayo Endoscopic Subscore
MPO: Myeloperoxidase
MPO-ANCA: Myeloperoxidase antineutrophil cytoplasmic antibody
NETs: Neutrophil extracellular traps
PR3: Anti-proteinase 3
PR3-ANCA: Anti-proteinase 3 antineutrophil cytoplasmic antibody
UC: Ulcerative colitis
5-ASA: 5-Aminosalicylic acid
